# Intelligence in Childhood and Atherosclerosis of the Carotid and Peripheral Arteries in Later Life: The Lothian Birth Cohort 1936

**DOI:** 10.1371/journal.pone.0125280

**Published:** 2015-04-27

**Authors:** Catharine R. Gale, Elizabeth Eadie, Avril Thomas, Mark E. Bastin, John M. Starr, Joanna Wardlaw, Ian J. Deary

**Affiliations:** 1 Centre for Cognitive Ageing & Cognitive Epidemiology, Department of Psychology, University of Edinburgh, Edinburgh, United Kingdom; 2 MRC Lifecourse Epidemiology Unit, University of Southampton, Southampton, United Kingdom; 3 Centre for Clinical Brain Sciences, University of Edinburgh, Western General Hospital, Edinburgh, United Kingdom; 4 Geriatric Medicine Unit, University of Edinburgh, Western General Hospital, Edinburgh, United Kingdom; 5 Department of Neuroradiology, Western General Hospital, Edinburgh, United Kingdom; Cardiff University, UNITED KINGDOM

## Abstract

**Objective:**

There is some evidence that people who score higher on tests of intelligence in childhood have lower carotid intima-media thickness and higher ankle brachial index in middle age. These findings need replicating in other, older populations. We investigated the prospective relationship between intelligence in childhood and atherosclerosis in the carotid and peripheral arteries at age 73 years.

**Methods:**

Participants were 713 members of the Lothian Birth Cohort 1936 whose intelligence was assessed at age 11 years. At age 73 years, carotid intima-media thickness and degree of stenosis were measured using ultrasound imaging; ankle-brachial index was measured using Doppler ultrasound.

**Results:**

There were no significant associations between intelligence at age 11 and measures of atherosclerosis at age 73. In age- and sex-adjusted analyses, for a standard deviation higher score in intelligence, intima-media thickness (x 10) was lower by 0.07 (-0.20, 0.06) mm and ankle brachial index (x 10) was lower by 0.09 (-0.24, 0.07); odds ratios for having carotid stenosis >25% or peripheral arterial disease were 0.98 (0.82, 1.16) and 1.05 (0.81, 1.36) respectively.

**Conclusion:**

In this study of people aged 73 years, higher childhood intelligence was not associated with reduced risk of atherosclerosis in the carotid or peripheral arteries.

## Introduction

There is consistent evidence that people who scored lower on tests of general cognitive ability in youth are more likely to develop coronary heart disease (CHD) as adults.[1–10] The explanation for these findings is unclear. They do not appear to be due to confounding by socioeconomic circumstances in early life. A few studies have been able to examine the potential mediating role of some established risk factors; in some of these studies such risk factors seem to account for very little of the association,[2, 6] but in others they account for a large part of it.[3, 9]

One potential mechanism linking intelligence in early life with later CHD risk might be atherogenesis. Atherosclerosis is a systemic, chronic inflammatory disorder that usually affects multiple parts of the arterial tree in the same individual. Measures of subclinical atherosclerosis such as carotid intima-media thickness (IMT) and ankle brachial index (ABI) correlate with severity and extent of coronary atherosclerosis[11, 12] and are consistently predictive of cardiovascular events.[13–15] Two recent longitudinal studies found evidence that lower intelligence in youth may increase susceptibility to atherogenesis. In the Newcastle Thousand Families Study, lower intelligence at age 11 years was associated with greater carotid IMT in men and women aged 49–51 years.[16] In the Vietnam Experience Study, male veterans who had scored lower on an intelligence test at conscription in early adulthood had a significantly lower mean ABI and were less likely to have peripheral arterial disease as indicated by an ABI ≤0.90 at around age 38 years.[17] However, since the major burden of CHD occurs in later life, these findings need replicating in other, older populations. There is also a need to examine the consistency of the relationship between intelligence in youth and atherogenesis in different parts of the arterial tree in the same sample. We used data from the Lothian Birth Cohort 1936 (LBC1936) to investigate the relationship between intelligence in childhood and atherosclerosis in the carotid and peripheral arteries at around age 73 years. One potential limitation in studying this relationship in people over age 70 is that lower intelligence is linked with earlier death[18] and with non-response[19, 20] so detecting an association may be difficult as individuals with lower intelligence may be under-represented in our sample.

## Methods

### Participants

The participants were members of the LBC1936.[21, 22] The study was set up to study cognitive ageing in surviving members of the Scottish Mental Survey of 1947. Between 2004 and 2007, individuals from the Lothian region of Scotland who might have taken part in the 1947 Survey were invited to participate in the LBC1936. The Lothian Community Health Index was used to identify 1936-born individuals, and media advertisements were used to inform people about the proposed study. The Lothian Community Health Index identified 3810 potential participants, and 3686 were invited to take part in the study. Overall, 2318 people responded, 1226 of whom were interested and eligible (97 of these people responded having seen the media advertisements). In total, 1,091 community-dwelling people took part in Wave 1 of the study when they were aged about 70 years. Wave 2 took place when participants were aged about 73 years; 866 people took part. Ethical approval was obtained from the Multi-Centre Ethics Committee for Scotland and Lothian Research Ethics Committee. All participants gave written informed consent.

### Measures

#### Intelligence in childhood

Most children born in 1936 and attending Scottish schools on 4^th^ June 1947 took the Moray House Test No 12, a test of general intelligence, when they were about age 11 years (mean 10.9, standard deviation 0.28) as part of the Scottish Mental Survey. The Moray House Test is a group-administered test with a predominance of verbal reasoning items and some numerical and spatial items. It was concurrently validated against the Terman-Merrill Revision of the Binet Scales.[23] The LBC1936 participants’ scores on the test were corrected for age at testing and converted to an IQ scale with mean of 100, standard deviation (SD) 15.

#### Atherosclerosis in the carotid and peripheral arteries

At the Wave 2 interview, participants were invited to come back at a later date for neck artery ultrasound imaging. Of the 866 people who took part in Wave 2, 820 agreed to have ultrasound imaging. This was performed on a Siemens Antares Premium Colour Doppler scanner (Siemens AG, Erlangen, Germany) with 7.5 MHz variable frequency probe by one of two experienced neurovascular ultrasonographers; all scans were cross-checked by a consultant neuroradiologist. Representative velocity readings were obtained of the common, internal and external carotid arteries.[24] Percent stenosis was quantified based on velocity criteria and luminal reduction, expressed in North American Symptomatic Carotid Endarterectomy Trial format.[25] Common carotid and carotid bulb intima-media IMT was measured manually with callipers. Mean IMT was calculated using the average of three measurements made over a 1 cm long segment of the common carotid artery and carotid bulb.[26]

During a physical examination carried out at Wave 2, brachial systolic pressure was measured in the right arm after 5 min of rest using a Doppler ultrasound probe and a random zero sphygmomanometer placed just above the elbow. Ankle systolic pressure was measured in the posterior tibial artery of the right leg using a Doppler ultrasound probe and a random zero sphygmomanometer with the cuff position just above the malleolus. The ABI was derived by dividing the systolic blood pressure in the ankle by that in the arm. Peripheral arterial disease was defined as having an ABI ≤ 0.90.

#### Covariates

We viewed father’s social class in childhood as a potential confounder of any association between childhood intelligence and later life measures of atherosclerosis.[27] At Wave 1, participants provided information on their father’s occupation when they were aged 11 years. Occupations were classified into six social class categories: professional, managerial, skilled non-manual, skilled manual, semi-skilled and unskilled. [28]

We viewed blood pressure, serum total cholesterol, glycosolated haemoglobin (HbA1c), smoking status, body mass index (BMI), and adult social class—all assessed at Wave 1—as potential mediators of any association between childhood intelligence and later life measures of atherosclerosis. Systolic and diastolic blood pressure were calculated as the average of three sitting readings taken using an Omron 705IT monitor. Blood samples (non-fasting) were taken during the participants' physical examination. Height and weight were measured with a portable stadiometer and electronic scales respectively. BMI was calculated as weight (in kilograms)/height (in metres)^2^. Adult socioeconomic position was derived from participants’ (or their spouses’) highest reported occupation and classified into categories as described above for father’s occupation.

### Statistical analysis

We used ordinary least squares regression to examine differences in ABI according to a standard deviation (SD) increase in intelligence at age 11 years. Robust regression was used to examine differences in carotid IMT in relation to intelligence to reduce the effect of influential outliers. Logistic regression was used to examine risk of having peripheral arterial disease (ABI ≤0.90) or carotid stenosis >25% in either the right or the left carotid arteries. Preliminary analyses showed that the relation between childhood intelligence and these measures of atherosclerosis did not differ by sex or age so we analysed men and women together and adjusted for sex and age. Analyses of carotid IMT, carotid stenosis and ABI are based on 713, 680 and 654 participants respectively who had data on childhood intelligence and father’s social class (representing 82%, 79% and 76% respectively of those who took part in the Wave 2 follow-up).

## Results

Table 1 shows the characteristics of the study participants.

**Table 1:** Characteristics of the study participants (n = 713).

	Mean (SD)	No (%)
Female		349 (49.0)
Age, years	72.5 (0.71)	
Father in professional/managerial social class in childhood		191 (26.8)
IQ at age 11 years	101.3 (14.9)	
Carotid IMT, mm	0.85 (0.20)	
Carotid stenosis>25%†		197 (29.0)
ABI*	1.09 (0.18)	
ABI≤90*		73 (11.7)

*based on 654 people

^†^based on 680 people

Table 2 shows the correlations between intelligence at age 11 years and carotid IMT, ABI, and maximum percent stenosis in either the right or left carotid artery. There were no statistically significant correlations between intelligence and any of these measures.

**Table 2:** Correlations between intelligence at age 11 years and measures of atherosclerosis at age 73.

	Intelligence	Carotid IMT	Maximum percent stenosis	ABI
**Intelligence**	-			
**Carotid IMT**	-0.056 (p = 0.14)	-		
**Maximum percent stenosis**	-0.057 (p = 0.13)	0.227 (p<0.001)	-	
**ABI**	-0.051 (p = 0.20)	-0.082 (p = 0.04)	-0.160 (p = 0.001)	-

Table 3 shows results of the regression analyses. In analyses adjusted for age and sex, there were no statistically significant associations between childhood intelligence and any measure of atherosclerosis in the carotid or peripheral arteries: for a standard deviation higher score in intelligence at age 11 years, intima-media thickness (x 10) was lower by 0.07 (-0.20, 0.06) mm and ankle brachial index (x 10) was lower by 0.09 (-0.24, 0.07); odds ratios for having carotid stenosis >25% or peripheral arterial disease were 0.98 (0.82, 1.16) and 1.05 (0.81, 1.36) respectively. Further adjustment for the potential confounding factor, father’s social class in childhood, had little effect on the size of these associations.

**Table 3:** Results of regression analyses of measures of atherosclerosis in the carotid and peripheral arteries in relation to intelligence in childhood.

	Carotid arteries		Peripheral arteries	
	Regression coefficient (95% CI for difference in carotid IMT (x10)	Odds ratio (95% CI) for carotid stenosis >25%	Regression coefficient (95% CI) for difference in ABI (x10)	Odds ratio (95% CI) for peripheral arterial disease
Intelligence, per SD increase, adjusted for:				
Sex and age	-0.07 (-0.20, 0.06)	0.98 (0.82, 1.16)	-0.09 (-0.24, 0.07)	1.05 (0.81, 1.36)
Sex, age and father’s social class in childhood	-0.07 (-0.19, 0.06)	0.97 (0.81, 1.15)	-0.08 (-0.23, 0.08)	1.08 (0.83, 1.41)

We repeated our analyses of childhood intelligence in relation to ABI and peripheral arterial disease excluding those with an ABI>1.4 (n = 32) in case arterial stiffness had produced falsely high levels, but this exclusion had little effect (data not shown).

In total, 9% of the participants had no data on father’s social class in childhood. To check whether the restriction of the analytical sample to those with data on father’s social class had affected our estimates of the size of the associations between childhood intelligence and the measures of atherosclerosis, we repeated our analyses without this restriction. Associations were very similar in size to those reported in Table 2.

Further adjustment for risk factors measured at Wave 1 that might potentially have mediated any association between childhood intelligence and later atherosclerosis, such as blood pressure, serum total cholesterol, smoking status, glycosolated haemoglobin, BMI, and adult social class had very little effect on the size of the associations (data not shown).

## Discussion

In this longitudinal study of older men and women whose intelligence had been assessed at age 11 years, we found no significant associations between intelligence in childhood and measures of atherosclerosis in the carotid or peripheral arteries at age 73 years. These findings contrast with results of two earlier studies in which lower intelligence in childhood or in early adulthood was associated with increased carotid IMT or lower ABI in middle-aged people.[16, 17]

One explanation for the inconsistency between the findings in the present study and those reported previously[16, 17] may lie with the age of our participants. Intelligence in childhood may be a more important risk factor for atherogenesis in adolescence and early to mid-adulthood than it is later in life. Some support for this comes from observations in another cohort that lower intelligence at age 11 was predictive of increased risk of cardiovascular disease, coronary heart disease and stroke in those who first experienced these events before age 65, but not in those who were over 65.[7] Data from repeated assessments of atherosclerosis in the same individuals over time are needed if we are to ascertain with accuracy whether the potential influence of childhood intelligence on atherogenesis diminishes with age. Another related explanation might be that those individuals who were particularly susceptible to the potential effect of childhood intelligence on atherogenesis—perhaps via its influences on health behaviours, blood pressure, or other cardiovascular risk factors[29–31]—were less likely to have survived to take part in this follow-up study. Unfortunately, we have no way of knowing whether this is the case because the cohort was only recruited when they were aged 70 so information on deaths that occurred prior to this age among people who would potentially have been eligible to participate is not available.

In view of the lack of association found in this study between childhood intelligence and measures of atherosclerosis in later life, we carried out a power calculation to assess whether our sample size was large enough to reliably show no association. To detect a correlation between childhood intelligence and IMT or ABI of -0.05 (the correlation found in our study) with 80% power using a one-sided 5%-level test would need a sample size of 2472, markedly larger than the sample of 713 on which our study was based.

Our findings suggest that higher intelligence in childhood is not associated with reduced atherogenesis in the carotid or peripheral arteries in people aged over 70. Further longitudinal studies are needed to establish the robustness of earlier observations linking lower intelligence in childhood or early adulthood with higher levels of atherosclerosis in the carotid or peripheral arteries in middle age and to investigate whether any such association persists with increasing age.
